# Ultrastructural and morphometrical study of preimplantation endometrium in superovulated mice treated with progesterone or Sildenafil

**Published:** 2013-10

**Authors:** Leila Roshangar, Jafar Soleimani-Rad, Bahman Rashedee, Hossein Mazochian, Behzad Nikzad, Sara Soleimani Rad

**Affiliations:** 1 *Neuroscience Research Center, Tabriz University of Medical Sciences, Tabriz, Iran.*; 2 *Drug Applied Research Center, Faculty of Medicine, Tabriz University of Medical Sciences, Tabriz, Iran.*; 3 *Department of Anatomical Sciences, Faculty of Medicine, Isfahan University of Medical Sciences, Isfahan, Iran.*; 4 *Medical Education Research Center, Tabriz University of Medical Sciences, Tabriz, Iran.*; 5 *Department of Neuroscience, Tabriz University, Tabriz, Iran.*; 6 *Faculty of medicine, Tabriz University of Medical Sciences, Tabriz, Iran.*

**Keywords:** *Implantation*, *Progesterone*, *Sildenafil*, *Mice*, *Uterine.*

## Abstract

**Background:** Endometrial development has an important role in blastocyst adhesion and implantation. During IVF cycles, endometrial development is enhanced by progesterone.

**Objective: **The aim of this study was to compare ultrastructural and morphometrical characteristics of mice uterine endometrium in natural cycle with those in superovulated cycles received progesterone or Sildenafil.

**Materials and Methods:** In This study, 60 female bulb/c mice were divided into 4 groups: a control and 3 experimental; gonadotropin, gonadotropin+ Sildenafil and gonadotropin+ progesterone. In experimental groups the mice superovulated mated. In the gonadotropin+ progesterone and gonadotropin+ Viagra groups, the mice respectively received 1mg progesterone and 3 mg Sildenafil citrate. Their uterine specimens were prepared for morphometrical and ultrastructural study. Height of the epithelial cells was measured, using motic software. Statistical analysis was performed using ANOVA.

**Results: **Microscopy revealed that in control group the cells had numerous apical microvilli and the height of the cells was 20.52±2.43 µm. In gonadotropin+ progesterone group, the granules were found in basal and apical portions and cellular height were 17.91±2.78 µm which were significantly shorter than in the control and gonadotropin groups (p<0.001). In this group, the apical membrane also contained pinopodes. In gonadotropin +Sildenafil group, the granules were found in both apical and basal portions and the height of the cells were 17.60±2.49 µm which were significantly shorter than in the control and gonadotropin groups (p<0.001). In this group, pinopodes appeared slightly extensive than the other groups.

**Conclusion:** It is concluded that superovulatory drugs in mice stimulate endometrial maturation but injection of Sildenafil is nearly more positive.

This article extracted from Ph.D. thesis. (Bahman Rashedee)

## Introduction

The low pregnancy rate in assisted reproductive technology (ART), is one of challenging issues which extensively is under investigation. Since the beginning of use of in vitro fertilization for treatment of infertility, the superovulatory methods and drugs and embryo culture media has progressed tremendously. However, the implantation rate in ART is still low (1, 2). It appears that low implantation rate is partly due to the interference of super ovulatory drugs with endometrial maturation and also lack of synchronicity between endometrial and blastocysts development (3-6).

In ART protocols, in addition to the use of superovulatory drugs, for acceleration of endometrial maturation, after oocyte collection, progesterone is also used. The effect of this hormone on endometrial receptivity is established and reported that progesterone could extends endometrial implantation period possibly by modulation of uterine cell proliferation and expression of genes involved in implantation (7-16). Sildenafil citrate (Viagra) is a drug that regarding to its balancing effect between contraction and relaxation, in smooth muscle, is primarily used for erectile dysfunction (17-25). 

There are evidence that Sildenafil citrate is a vasodilator which act by increasing intracellular nitric oxide (NO) and it also enhances vasodilatory effect of NO by inhibiting the degradation of cGMP (26-28). Similar actions are proposed for Viagra in the myometrium and it is shown that vaginal use of Sildenafil improves uterine artry blood flow (29-31). Therefore, it appears that Viagra by increasing endometrial circulations could facilitate endometrial development and receptivity (32). Morphological characteristics of endometrium, especially endometrial luminal epitheliumat preimplantation period, arecorrelates well with its receptivity at implantation period (6). 

Consequently, it could be used as valuable criteria for evaluating endometrial development. The aim of the present study was to compare morphological characteristics of endometrium such as extention of pinopods, height of endometrical epithelial cells and location of secretory granules as an induction of endometrical development in superovulated mice after receiving progesterone or Viagra and immediately before implantation.

## Materials and methods

**Animals and treatment**

In this experimental study, 60 adult female bulb/c mice with average weight of 25-30 gr and 30 adult male mice were used. The mice housed at room temperature under a standard condition, light cycle (12-h light/dark) and free access to food and water. The ethical committee of Tabriz University of Medical Sciences (TUMS) approved all aspects of this study following laboratory practice guidelines. The female mice were divided into 4 groups of 15 as: control, gonadotropin, gonadotropin+ progesterone and gonadotropin+ Sildenafil. 

Gonadotropin was purchased from IBSA institute biochimique SA, Switzerland and Sildenafil was purchased from Rooz Daroo Co. Tehran, IRAN. Except in control group, in other experimental groups the mice received 7.5 IU hCG as intraperitoneal injection and 48 hours later 7.5 IU hMG. Then in all groups, two female mice at their oestrous cycle were put with one male mouse in a cage for mating. The presence of vaginal plug in the next morning was designated as 0 day post coitum (d.p.c). 

In the gonadotropin+ progesterone and gonadotropin+ Sildenafil groups, the mice respectively received 1mg progesterone and 3mg Sildenafil citrate at 24 , 48 and 72 hours after HMG injection. 96 hours after HMG injection using 23 gauge needles, the mice in experimental groups together with control mice were sacrificed and uterine specimens were obtained and processed for microscopic evaluation.

**Light microscopy**

For having uterine specimens exactly at preimplantation stage, the specimens only from those that their uterine contained blastocyst were fixed in 10% formalin, embedded in paraffin and 5 µm thick sections were stained with H & E and PAS and studied with light microscope. For measurement of hight of epithelial cells, the images were transferred to monitor, using Motic image analyzer system plus 2, and the measurement was carried out at higher magnificationas which was shown in previous study (33).

**Transmission electron microscopy**

Uterus from both control and experimental group fixed in 2.5% glutaraldehyde (Proscitech, Thuringowa, Australia) in phosphate buffer and processed for transmission electron microscopy at Tabriz University of medical sciences, drug applied and research center, histology department EM facility. 

Samples were postfixed in 1% OsO_4_ (TAAB, Berkshire- Uk) dehydrated through an ethanol series, equilibrated in propylene oxide, and embedded in Araldite (Proscitech, Thuringowa, Australia). Thin sections were stained with uranyle acetate and lead citrate. The specimens were studied with LEO 906 transmission electron microscope for pinopodes morphology of nuclei and localization of granules. The images recorded with AMT advantage plus CCD camera.

**Statistical analysis**

The values in every time point from control and treated groups were analyzed with ANOVA using SPSS 13 and the level of p<0.05 was considered significant. 

## Results

**Figure 1:**
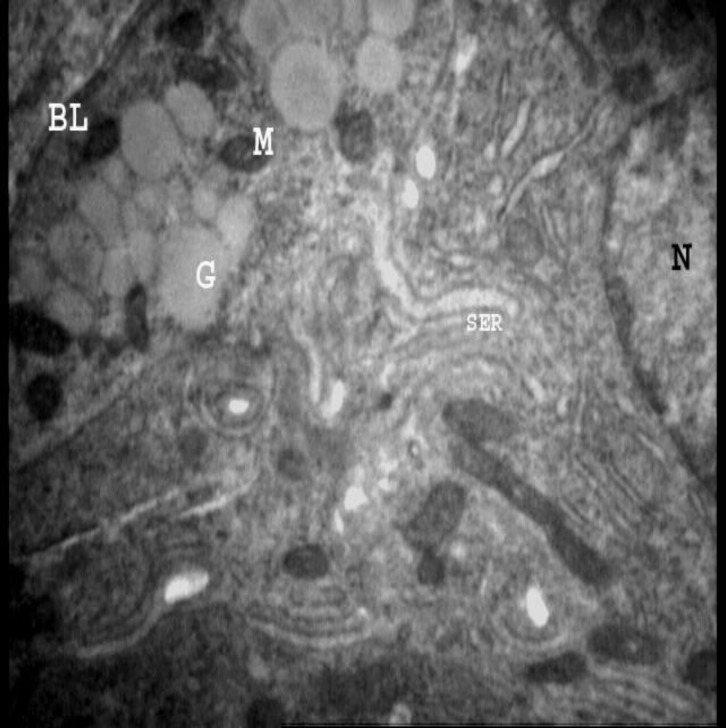
Electron micrograph from epithelial cell of endometrium in control mouse. Note basal lamina (BL), Nucleus (N), mitochondrium (M), granules (G), smooth endoplasmic reticulum (SER). 13700 X

**Figure 2:**
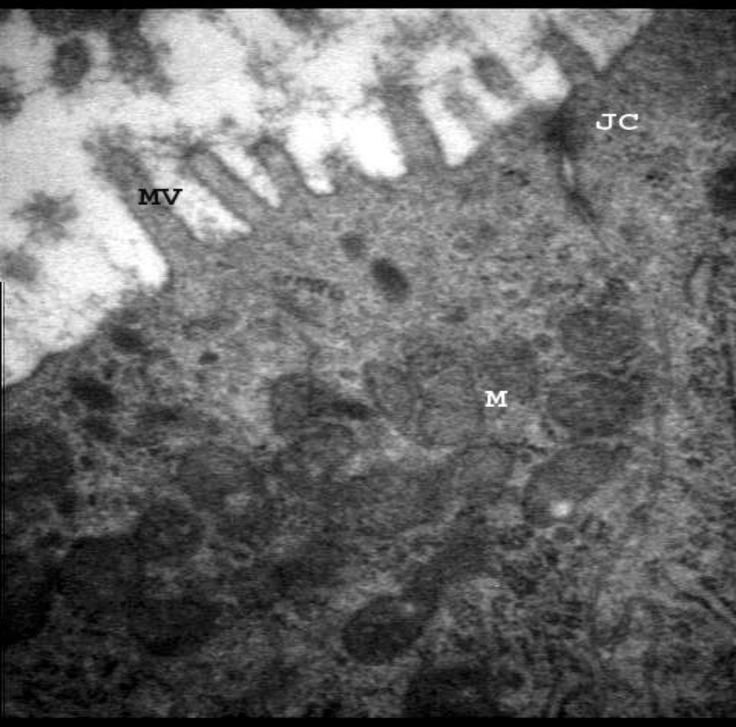
Electron micrograph from endometrial epithelial cells from control mouse. Note, Junctionalcomplex (JC), mithochondria (M), microvilli (MV). 13500X

Light microscopic study revealed that the cells of endometrial luminal epithelium were tall columnar in both control and those that only received HMG and HCG. But the cells were low columnar in those groups that received progesterone or Viagra in addition to superovulatory drugs. On the other hand, in the first two groups, PAS positive granules were restricted to sub nuclear and supranuclear regions, while in the experimental groups which were received progesterone or Viagra, they were dispersed in the cytoplasm. Electron microscopy confirmed the light microscopic findings and showed that in control group the endometrial luminal cells contained euchromatic nuclei, several basal granules, numerous mitochondria and a well-developed rough endoplasmic reticulum (Figure 1, 2). 

**Figure 3:**
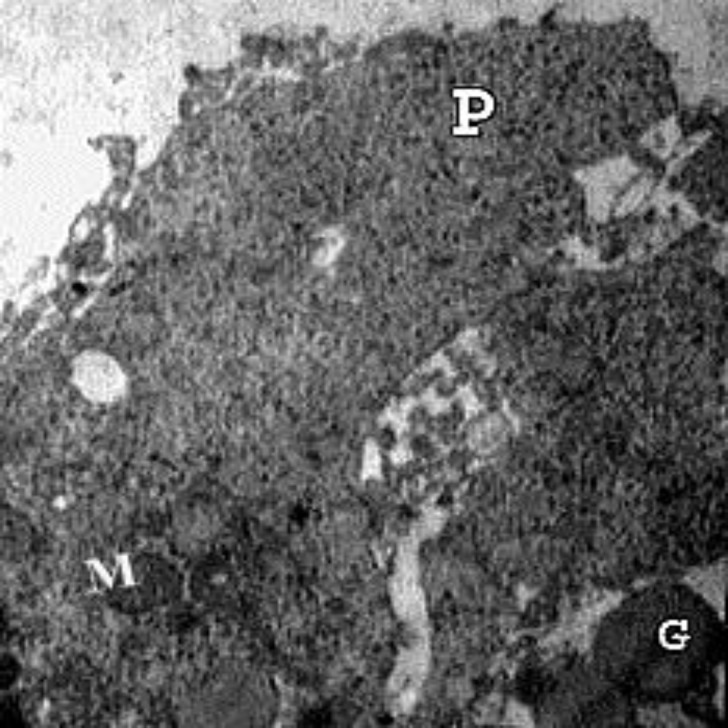
Electron micrograph from endometrial epithelial cells from the group received gonadotropin. The micrograph shows Pinopods (P), mithochondria (M), and numerous apical granules (G).6646X

**Figure 4:**
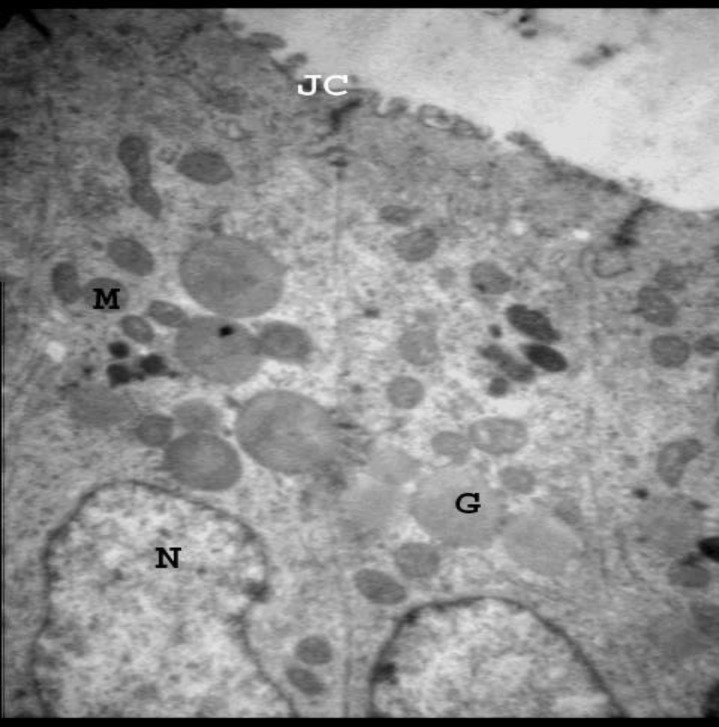
Electron micrograph from endometrial epithelial cells from experimental group received gonadotropin+progestrone. Note, Junctional complex (JC), several mithochondria (M), Nucleus (N) and numerous supranuclear granules (G). 13500X

**Figure 5:**
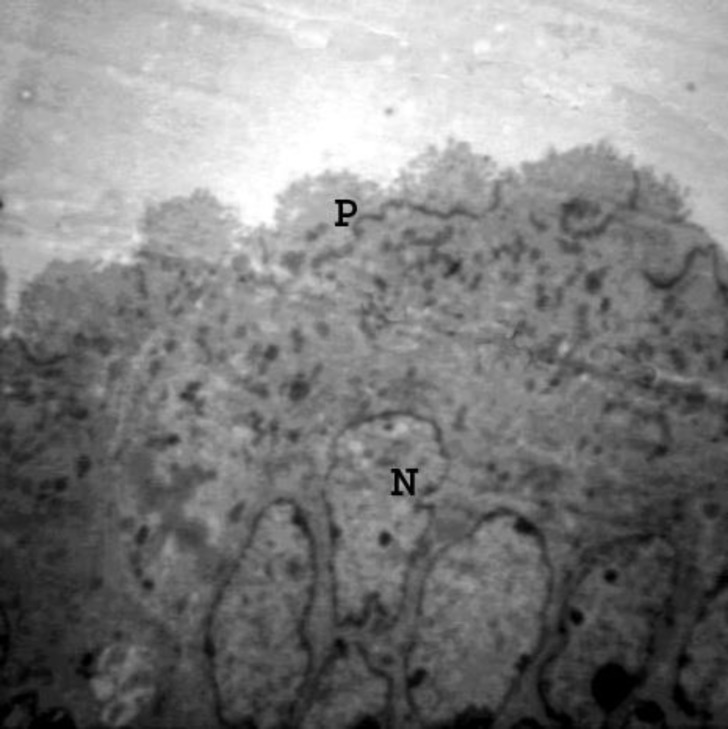
Electron micrograph from endometrial epithelial cells from experimental group received gonadotropin+Viagra. Note, the presence of extensive pinopods (P) and euchomatic Nuclei (N). 9500X

**Figure 6:**
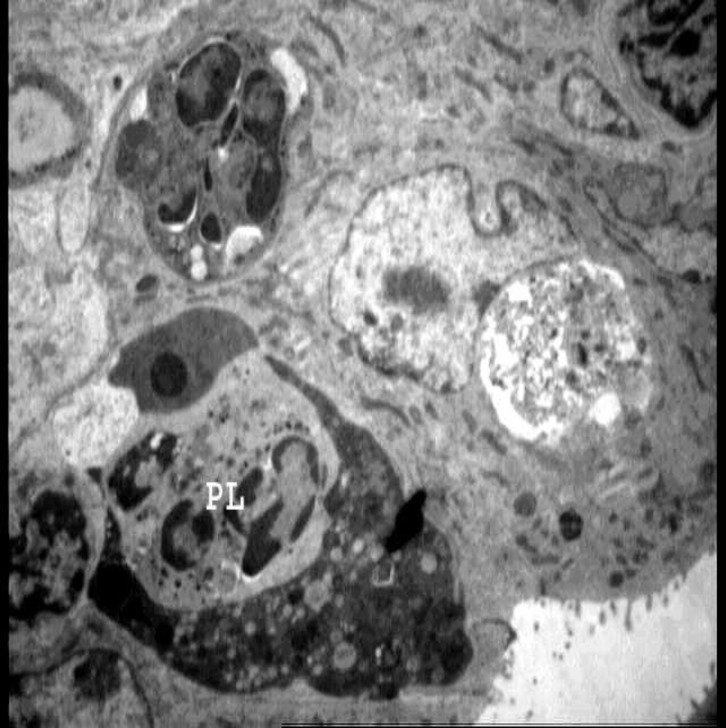
Electron micrograph from endometrial epithelialand stromal cells from experimental group received gonadotropin+ progestrone. Note, the presence of intracellular phagolysosomes. (G) 135

The cells had also several apical microvilli (Figure 2). The HMG-HCG group had euchromatic nuclei and granules were more localized at apical region and pinopodes were frequent at luminal surface (Figure 3). In the group that received progesterone, in addition to HMG+HCG, granular localization was similar to HMG-HCG group but apical microvilli were as in control group (Figure 4). In the group that received Viagra, instead of progesterone, the apical granules and pinopodes were much similar to those in superovulated group (Figure 5) and several phagolysosomes were present in the stromal cells (Figure 6). The results of morphometric study are summarized in table I. 

As it is shown in the table the height of the endometrial epithelial cells in the control group was 20.52±2.43µm and in gonadotropin group was 22.64±1.64. In gonadotropin+ progesterone group, the heights of the cells were 17.91±2.78 µm and in gonadotropin+ sildenafil was 18.23±0.71. The differences were statisticaly significant (p<0.001.

**Table I:** The height of the endometrial epithelial cells (μm) in control and experimental groups

**Groups**	**Control**	**Gonadotropin**	**Gonadotropin + progesterone**	**Gonadotropin+ Sildenafil**
height of the endometrial cells	20.52±2.43	22.64± 1.64*	17.91±2.78 *	18.23±0.71*

* : The values indicated with mean±SD were significantly (p<0.001) different from control group.

## Discussion

The results of the present study showed that the height of the endometrial luminal epithelium in control and HMG-HCG groups were higher than the height of cells in gonadotropin+ progesterone or Viagra groups. This is probably due to the stimulatory effect of gonadotropin on folliculogenesis which could result in higher estrogen level. The hypothesis of progesterone injection in IVF cycles is that, endometrial epithelial maturation is facilitated by progesterone secretion during secretory phase. However, it appears that early progesterone injection interfere with the effect of estrogen and affect the height of the cells. 

In the support of our finding it is shown that estrogen results in hyperplasia, hypertrophy and increasing of cell height (34). On the other hands high level of progesterone is accompanied by decreased cellular height in endometrial luminal epithelium (35-40). Ultrastructural studies showed that apical granules and pinopodes on luminal surface of endometrial epithelium were more frequent in gonadotropin and gonadotropin+ Viagra groups in comparison to control and progesterone groups. Taking the above morphological features as indications of endometrical maturation, it is indicated that gonadotropin accelerates the endometrial maturation and receptivity.

While progesterone slightly suppresses the stimulatory effect of gonadotropins (41). Viagra has almost not any negative effect on gonadotropin-induced changes in endometrial epithelium. Similarly it is shown that superovulation protocol accelerates development of implantation window (42). The results of the present study are unique in that, the morphological changes of the endometrium are studied immediately, before implantation. 

A condition that its study in human is impossible (43-45). To make sure that all the samples are from the same stage of development. The uterine was flashed and specimens was obtained only from those that blastocyst could be collected.

## Conclusion

It is concluded that administration of Sildenafil rather than progesterone in superovulation protocol may have positive effect on implantation. However further studies and detection of molecules, involved in endometrial receptivity and implantation, would be needed to come to a definite conclusion.
